# NRF2 Antioxidant Response and Interferon-Stimulated Genes Are Differentially Expressed in Respiratory-Syncytial-Virus- and Rhinovirus-Infected Hospitalized Children

**DOI:** 10.3390/pathogens12040577

**Published:** 2023-04-09

**Authors:** Leonardo Sorrentino, Walter Toscanelli, Matteo Fracella, Marta De Angelis, Federica Frasca, Carolina Scagnolari, Laura Petrarca, Raffaella Nenna, Fabio Midulla, Anna Teresa Palamara, Lucia Nencioni, Alessandra Pierangeli

**Affiliations:** 1 Laboratory of Virology, Department of Molecular Medicine, Sapienza University, 00185 Rome, Italy; 2 Department of Public Health and Infectious Diseases, Laboratory Affiliated to Istituto Pasteur Italia-Fondazione Cenci Bolognetti, Sapienza University, 00185 Rome, Italy; 3 Department of Maternal Infantile and Urological Sciences, Sapienza University, 00185 Rome, Italy; 4 Department of Infectious Diseases, Istituto Superiore di Sanità, 00161 Rome, Italy

**Keywords:** respiratory syncytial virus, human rhinovirus, interferon, NRF2, antioxidant response

## Abstract

Respiratory diseases caused by respiratory syncytial virus (RSV) and human rhinovirus (HRV) are frequent causes of the hospitalization of children; nonetheless, RSV is responsible for the most severe and life-threatening illnesses. Viral infection triggers an inflammatory response, activating interferon (IFN)-mediated responses, including IFN-stimulated genes (ISG) expression with antiviral and immunomodulatory activities. In parallel, the reactive oxygen species (ROS) production activates nuclear factor erythroid 2-related factor 2 (NRF2), whose antioxidant activity can reduce inflammation by interacting with the NF-kB pathway and the IFN response. To clarify how the interplay of IFN and NRF2 may impact on clinical severity, we enrolled children hospitalized for bronchiolitis and pneumonia, and measured gene expression of type-I and III IFNs, of several ISGs, of NRF2 and antioxidant-related genes, i.e., glucose-6-phosphate dehydrogenase (G6PD), heme oxygenase 1 (HO1), and NAD(P)H dehydrogenase [Quinone] 1 (NQO1) in RSV- (RSV-A N = 33 and RSV-B N = 30) and HRV (N = 22)-positive respiratory samples. NRF2 and HO1 expression is significantly elevated in children with HRV infection compared to RSV (*p* = 0.012 and *p* = 0.007, respectively), whereas ISG15 and ISG56 expression is higher in RSV-infected children (*p* = 0.016 and *p* = 0.049, respectively). Children admitted to a pediatric intensive care unit (PICU) had reduced NRF2 expression (*p* = 0.002). These data suggest, for the first time, that lower activation of the NRF2 antioxidant response in RSV-infected infants may contribute to bronchiolitis severity.

## 1. Introduction

Respiratory syncytial virus (RSV) is the main cause of bronchiolitis [1,2], an illness characterized by acute inflammation and respiratory distress in infants, and pneumonia in older children [3]. RSV is endemic worldwide and infects almost all children by 2 years of age, causing severe disease only in a small percentage of those infected [2]. Reinfections are frequent as the immune response to RSV is not permanent; consequently, RSV may cause severe pneumonia in older adults [4].

Despite being more frequently associated with the common cold, rhinoviruses (HRV) have been recognized as agents of lower respiratory tract illnesses including bronchiolitis in infants [5], and pneumonia cases in children and in the elderly [6]. Moreover, HRVs represent the main risk factor associated with recurrent wheezing illnesses in young children and exacerbations of chronic airway diseases [7].

RSV and HRV are the most frequent causes of hospitalization of infants and children up to 3 years of age for bronchiolitis [5] and pneumonia [8]; nonetheless, RSV causes the most severe and life-threatening infections [8,9].

Upon infection by respiratory viruses, naso-pharyngeal mucosal cells activate innate responses, relying on the production of type I and III interferons (IFN) [10,11] and on the stimulation of neutrophils and monocytes. In turn, IFNs activate hundreds of IFN-stimulated genes (ISGs) with antiviral and immunomodulatory activity [10,11]. A prolonged and/or dysregulated production of ISGs and other innate immunity components can contribute to immunopathology, causing damage to the infected mucosa [12,13]. In fact, clinical severity in respiratory infections can be associated with IFN genes overexpression [14,15], and/or to an excess of inflammatory response [3,12,16].

Viral infections determine oxidative stress in host cells and this condition has been related to an increase in viral replication [17,18]. As a consequence, the reactive oxygen species (ROS) production activates pathways that respond to the oxidative stress in an attempt to prevent tissue damage. A crucial role in the antioxidant response is played by the activation of the nuclear factor erythroid 2-related factor 2 (NRF2) [17,19], a key transcription factor that, upon release, translocates into the nucleus and activates the expression of antioxidant enzyme (AOE) genes [20]. Among AOE, glucose-6-phosphate dehydrogenase (G6PD) is indispensable to maintain the cytosolic pool of NADPH and, thus, the cellular redox balance [21], while heme oxygenase 1 (HO1) is an important anti-inflammatory target of NRF2 [22], and NAD(P)H dehydrogenase [Quinone] 1 (NQO1) is a crucial enzyme involved in the detoxification of quinones and redox signaling [23]. Besides transcriptional activation of AOE, NRF2 is able to reduce inflammation and tissue damage, repressing pro-inflammatory gene expression [24], and limiting the IFN response by interacting with several components of innate immune signaling [25,26].

In recent years, several works reported a modulation of NRF2 during infections. Among this evidence, NRF2 downmodulation has been observed during enterovirus 71, rotavirus, hepatitis C, and HIV infections [27]. Interestingly, NRF2 suppression also occurs during respiratory infections, such as the recent SARS-CoV-2 infection; nonetheless, NRF2 is able to modulate the host response against these pathogenic viruses [18,28,29]. In particular, studies demonstrate that RSV is able to downregulate NRF2 levels [30,31] during infection of human airway epithelial cells [32] and in mouse models [33]. The same group demonstrated that, in nasopharyngeal secretions of RSV in severe bronchiolitis cases, oxidative stress markers were elevated and AOEs were reduced [34]. Moreover, the protective role of NRF2 in RSV has been documented in mouse models [30] and an increase in NRF2 activation and subsequent AOE expression significantly reduced the oxidative stress during RSV infection by using in vitro and in vivo models [32,35]. However, NRF2 expression has not been directly measured in human RSV infections, yet.

HRV infections cause oxidative stress and the induction of type I and III IFNs in a complex balance of these responses, directed to ensure antiviral actions and airway protection [36,37]. A direct role of HRV proteins in modulating NRF2 has not been demonstrated yet. Interestingly, in HRV experimental infections of primary human airway epithelial cells, those of nasal origin showed a more abundant IFN response, whilst primary bronchial cells exhibited lower IFN expression and a higher NRF2-mediated antioxidant response, probably to preserve bronchial cells from IFN-mediated immunopathology [37].

In order to provide more insights on the activation of the IFN response and on the role of the antioxidant defense in virus-induced respiratory pathology, we sought to evaluate the transcriptional levels of type I/III IFNs and ISGs, NRF2 and AOEs, in samples from an age-homogeneous group of hospitalized children, comparing RSV and HRV infections.

## 2. Materials and Methods

### 2.1. Study Subjects

Children up to 24 months of age, born at term, non-low birth weight, and previously healthy, hospitalized with bronchiolitis, or other acute respiratory infections (ARI), at the Pediatric Department, “Sapienza” University of Rome during the 2017–18, 2018–19, and 2019–20 winter seasons, were enrolled after the informed consent obtained from parents. Clinical and demographic data were collected and, in line with confidentiality requirements, the database was anonymized; the study was approved by the ethic committees of the Sapienza University Hospital Policlinico Umberto I, (Prot. 107/12). Bronchiolitis was strictly defined as the first episode of acute lower airway infection, with respiratory distress and diffuse crackles on auscultation in infants less than 12 months of age. A clinical severity score ranging 0–8 was assigned to bronchiolitis cases at hospital admission, according to respiratory rate, oxygen saturation on room air (SaO_2_), presence of retractions, and ability to tolerate oral feeding, as previously described [9]. Blood samples from patients and respiratory samples from healthy controls could not be obtained because of ethical concerns.

### 2.2. Gene Expression Measurement

Nasopharyngeal washings (NPW) collected within the first 24 h after hospital admission were tested for 14 respiratory viruses by molecular methods [9]; RSV-positive samples were subsequently genotyped by sequencing, as previously described [15]. The remaining aliquots of NPWs were centrifuged, cell pellet resuspended in guanidine isothiocyanate reagent, and frozen at −80 °C for subsequent gene expression analysis [38]. The mRNA copy numbers of the key genes of the cellular antioxidant pathway (NRF2, G6PD, HO1, and NQO1), IFN beta, the type III IFNs (lambdas 1–3), and the ISGs (ISG15, ISG56, and the MxA) were measured by quantitative RT-PCR assays, in co-amplification with the beta-glucuronidase gene to normalize the amount of total RNA, using the threshold cycle relative quantification (the 2^−ΔCt^ method) [38]. The gene expressions of NRF2, G6PD, HO1, and NQO1 were determined by qPCR reactions with the SensiFAST SYBR NO-ROX Kit (Meridian Bioscience) according to the manufacturer’s protocol, using the following primers: NRF2 forward 5′-CGTTTGTAGATGACAATGAGG-3′ and reverse 5′-AGAAGTTTCAGGTGACTGAG-3′; G6PD forward 5′-CGTCACCAAGAACATTCAC-3′ and reverse 5′-GGAGATGTGGTTGGACAG-3′; HO1 forward 5′-CTCAAACCTCCAAAAGCC-3′ and reverse 5′-TCAAAAACCACCCCAACCC-3′; NQO1 forward 5′-GGAGAGCACTGATCGTACTGGC-3′ and reverse 5′-GGATACTGAAAGTTCGCAGGG-3′. The amplification was performed using the iQ5 BIO-RAD multicolor real-time PCR detection system; data were normalized relative to beta-glucuronidase.

### 2.3. Statistical Analysis

For categorical variables, Pearson’s chi-square was used. mRNA expression values (non-normally distributed data) were compared using the non-parametric Mann–Whitney test between two groups or Kruskal–Wallis among three groups. The Jonckheere–Terpstra (JT) test, a rank-based non-parametric test, was also used to determine significant trends in gene expression levels among the virus groups ordered by clinical severity in RSV-A, RSV-B, and HRV. Spearman’s ρ coefficients were calculated to assess correlations between study genes’ expression and age in months. The significance was fixed at the 5% level; the analysis was performed with SPSS v.27.0 for Windows. R-software was used to display graphic representation of correlation coefficients.

## 3. Results

### 3.1. RSV and HRV Detection and Clinical Diagnosis in the Study Groups

Previously healthy children up to 24 months of age, hospitalized for respiratory illness, were enrolled in this study if they tested positive for RSV (N = 63) or HRV (N = 22) in a single infection (Table 1). RSV-positive samples were subtyped into RSV-A (N = 33) and RSV-B (N = 30). The 85 enrolled patients had a mean age of 4.93 months and 52 (61.2%) were male; at hospital admission, 77 children were diagnosed with bronchiolitis, while 8 had pneumonia. Seven children (six RSV-A and one RSV-B) required admission in the paediatric intensive care unit (PICU). According to the bronchiolitis severity score, children were divided in three groups, low severity score (N = 29), intermediate severity score (N = 24), and high severity score (N = 17); as evidenced in Table 1, worst severity scores were more frequently attributed to RSV-infected children.

### 3.2. Different Gene Expression Based on RSV/HRV Status and Clinical Data

From residual NPW samples, the expression levels of genes related to antioxidant and IFN-pathways were calculated and compared on the basis of patients’ characteristics and the different infecting viruses; results are reported in Table 1. No significant difference is found when comparing expression levels of the studied genes by sex; moreover, transcript values do not correlate with patients’ age.

Levels of gene expression were then compared between infants infected with RSV and HRV, as reported in Table 1. Transcript levels of the NRF2 and HO1 genes are significantly higher in the HRV-infected patients with respect to RSV-infected infants (*p* = 0.012 and *p* = 0.007, respectively). Although not significant, a slight reduction in NQO1 expression is also found in RSV compared to HRV. Concerning the IFN-coding genes, they do not differ significantly between groups, whereas the ISG-coding genes, ISG15, ISG56, and MxA, are significantly more activated in the RSV-infected group than in the HRV-infected group (*p* = 0.006, *p* = 0.014, *p* = 0.028, respectively).

**Figure 1:**
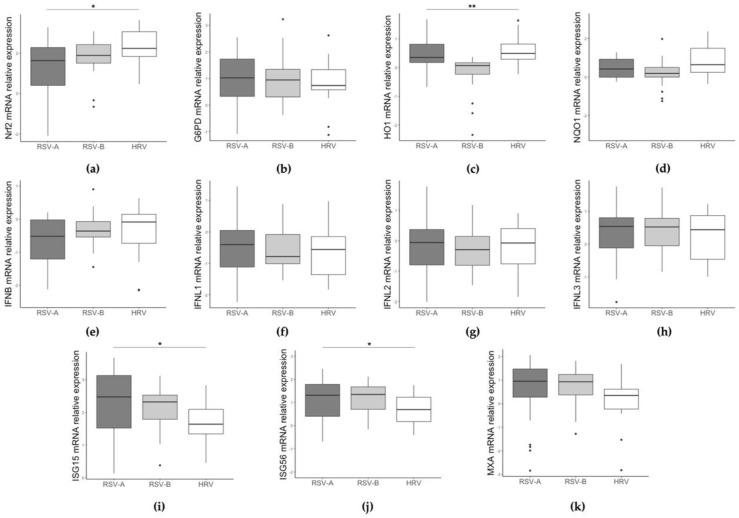
Comparison of NRF2 (**a**), G6PD (**b**), HO1 (**c**), NQO1 (**d**), IFN beta (**e**), IFNL1 (**f**), IFNL2 (**g**), IFNL3 (**h**), ISG15 (**i**), ISG56 (**j**), and MXA (**k**) gene expression among RSV-A, RSV-B, and HRV infected children. Data are shown as Log_10_ (2^−ΔCt^). * *p* < 0.05, ** *p* < 0.01.

Next, stratifying samples in three groups (RSV-A, RSV-B, and HRV), NRF2 and HO1 expression significantly differs among groups (Figure 1a,c, *p* = 0.014 and *p* = 0.001, respectively). NRF2 values are higher in HRV-infected children and lower expression is found in the RSV-A group. With regard to antioxidant genes expression, HO-1 is significantly downregulated in RSV-B-infected children compared to the HRV group; similarly, a weak reduction in NQO1 expression is found in RSV-B-infected children compared to the HRV group. ISG15 and ISG56 expression levels are also significantly different among the three groups (Figure 1i,j, *p* = 0.016 and *p* = 0.049, respectively), with the lower values in HRV-infected group and the higher in the RSV-A infected children.

**Figure 2:**
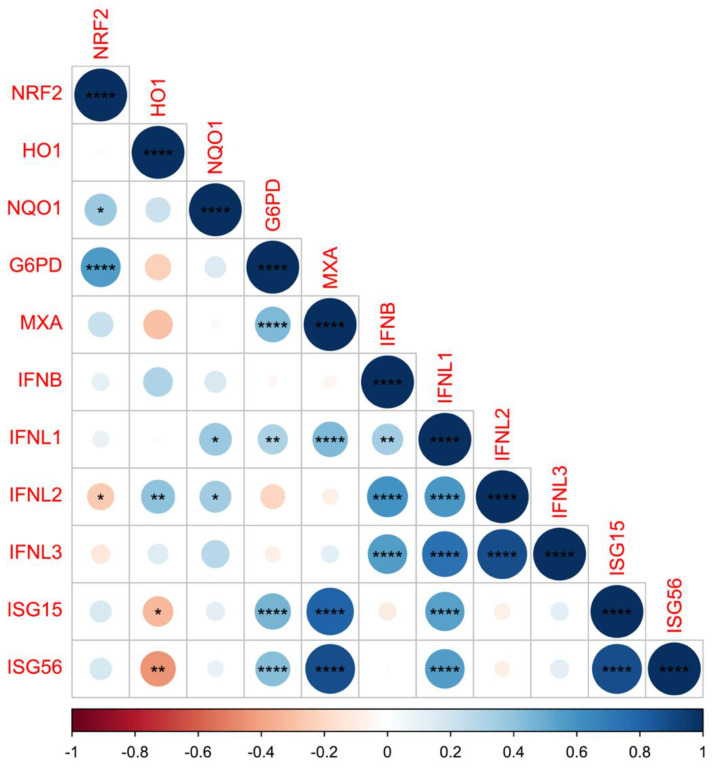
Graphical display of the correlation matrix for NRF2, HO1, NQO1, G6PD, MXA, IFN beta, IFNL1, IFNL2, IFNL3, ISG15, and ISG56 levels of expression using the ‘corrplot’ package of the R-software. Spearman’s rank correlations are represented by red (negative correlation) and blue (positive correlation) color gradients. * *p* < 0.05, ** *p* < 0.01, **** *p* < 0.0001.

Interestingly, HO1 expression is negatively correlated with ISG15 and ISG56 expression (r = −0.321, *p* = 0.038, and r = −0.441, *p* = 0.003, respectively) (Figure 2).

**Figure 3:**
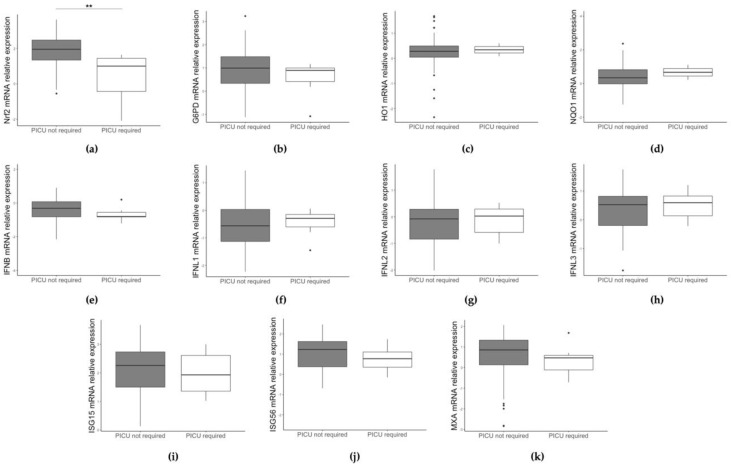
Comparison of NRF2 (**a**), G6PD (**b**), HO1 (**c**), NQO1 (**d**), IFN beta (**e**), IFNL1 (**f**), IFNL2 (**g**), IFNL3 (**h**), ISG15 (**i**), ISG56 (**j**), and MXA (**k**) gene expression between children admitted and children not admitted to PICU. Data are shown as Log_10_ (2^−ΔCt^). * *p* < 0.05, ** *p* < 0.001.

Study results were then analyzed with respect to patients’ clinical data. Interestingly, NRF2 expression (but not that of the other studied genes) is significantly lower (*p* = 0.002) in infants admitted to the PICU (Figure 3a).

**Figure 4:**
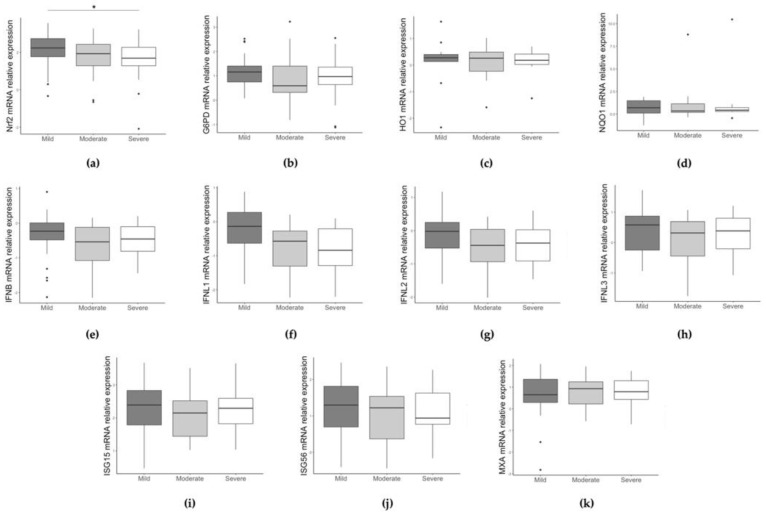
Comparison of NRF2 (**a**), G6PD (**b**), HO1 (**c**), NQO1 (**d**), IFN beta (**e**), IFNL1 (**f**), IFNL2 (**g**), IFNL3 (**h**), ISG15 (**i**), ISG56 (**j**), and MXA (**k**) gene expression between children grouped by severity score. Data are shown as Log_10_ (2^−ΔCt^). * *p* < 0.05, ** *p* < 0.001.

Next, we performed the analysis of gene expression levels among patients stratified in three severity score groups. NRF2 expression shows the tendency (KW test, *p* = 0.098; JT test, *p* = 0.031) to be lower in the infants with higher severity scores (Figure 4a).

## 4. Discussion

Both RSV and HRV cause an elevated disease burden and hospitalizations in small children; nonetheless, bronchiolitis and pneumonia cases caused by RSV are generally more severe and life-threatening than those caused by HRV [3,5,9]. The contribution of immunopathology to severe RSV infections has been intensively investigated [13,14,15,16]; however, the relative role of host antiviral and inflammatory responses, as well as the modulation of antioxidant response in worsening the disease course, is still controversial. Here, we compared NRF2 and its related AOE gene expression in nasopharyngeal washing from RSV- and HRV-infected children, and evaluated the relationships with the IFN-mediated antiviral response and disease severity. For the first time, to the best of our knowledge, we found that NRF2 and HO1 were expressed less in the more severely ill RSV-positive infants, in the presence of enhanced levels of ISGs, with respect to HRV-infected cases.

These results are in keeping with studies demonstrating that RSV infection induces NRF2 degradation in cellular and mouse models [32,33]. These pivotal studies provide a mechanistic explanation of RSV-mediated NRF2 downregulation, but no study directly measured NRF2 expression in patients’ infected cells.

In normal conditions, NRF2 is retained in the cytoplasm in association with Kelch-like ECH-associated protein 1 (KEAP1), which promotes NRF2 ubiquitination and subsequent degradation by proteasomes [17,39]. In the presence of augmented ROS production, electrophile-induced modifications of the KEAP1/NRF2 complex lead to the inhibition of KEAP1, and NRF2 escapes degradation and translocates to the nucleus, where it binds to the antioxidant-response element (ARE) [17,40]. NRF2 binding to AOE promoters results in the expression of numerous AOEs, encoding proteins involved in cellular redox homeostasis, detoxification, macromolecular damage repair, and metabolism [17].

Several viruses, including respiratory ones, have evolved strategies to manipulate the NRF2 pathway, both positively and negatively [17,18,25,27]. Indeed, we recently found a downmodulation of NRF2 expression at transcriptional and translational levels during influenza virus replication, to maintain oxidative stress useful for the activation of redox-sensitive pathways that favor viral replication [18]. Other authors demonstrated a downregulation of NRF2 antioxidant gene expression and IFN-inducible genes in patients with severe COVID-19 [29]. In agreement, in our study, we observed a relatively low transcriptional activation of NRF2 and HO1 expression in severe RSV bronchiolitis. We did not analyze antioxidant protein levels because respiratory samples obtained from infected patients were not sufficient to detect proteins. Nonetheless, there is a biological meaning in measuring NRF2 transcriptional levels at the time of hospitalization that occurs several days after infection. In fact, it has been reported that NRF2 can positively regulate its own gene transcription at a later time after infections [26,34].

NRF2 has been described as an important regulator of the IFN type I response, by repressing the antiviral cytosolic sensing such as mitochondrial antiviral signaling (MAVS) and the expression of the adaptor STING [25]. Previous studies demonstrate that NRF2 targets the IRF3-dependent type-I IFN induction [26]; in addition, a recent paper showed that in macrophages, NRF2 may also directly downregulate the mRNA and protein expression of the type I IFN receptor and downstream ISGs production [41]. Indeed, the use of itaconate that activates NRF2, affects the Janus kinase 1 (JAK1) and signal transducer and activator of transcription 1 (STAT1) phosphorylation, important in IFN type I signaling [26]. Here, we observed a lower NRF2 activation, associated with higher expression of ISGs, in the RSV-positive children compared to the HRV-positive children. Obviously, a causal association between NRF2 and ISGs cannot be proven in this in vivo study, but the finding that HO1 expression negatively correlates with ISG15 and ISG56 strongly supports the concept that the impairment of the NRF2-mediated antioxidant pathway leads to an increased inflammatory response. Accordingly, it is tempting to speculate that lower levels of NRF2 may also cause an uncontrolled ISGs production that, in addition to the decrease in the antioxidant defense, may contribute to bronchiolitis severity. Indeed, RSV-infected children unable to control ISGs’ level are affected by a more severe disease, as several studies, including ours, demonstrate [14,15,38].

We are aware of some limitations in our study: firstly, the lack of samples from healthy controls and the relatively low number of nasopharyngeal cells obtained from residual diagnostic specimens limited the analysis of several genes involved in these pathways, including cytokines and chemokines promoted by the IFNs and those triggered by the antioxidant-related response. Therefore, to clarify the relationships of the antioxidant defense with the IFN response induced by RSV and HRV, we focused our attention on NRF2 and three NRF2-regulated genes (G6PD, HO1, and NQO1), on the type I IFN beta, the type III IFNs, and three ISGs, well-known markers of type I and III IFNs activation that are highly RSV- and HRV-inducible [14,15,38,42]. Moreover, we acknowledge that the RNA sequencing techniques would give a more complete picture of the complex innate immune response activation. Nevertheless, real-time PCR assays performed with gene-specific probes are reliable, not expensive, quantitative tests that allow us to analyze the expression of twelve cellular genes from residual diagnostic samples.

In conclusion, we report, for the first time, decreased NRF2 and HO1 levels and an inverse correlation with ISGs in RSV-infected, hospitalized children that shows higher disease severity than HRV-infected patients, thus, suggesting the contribution of NRF2 dysregulation to the severity of such viral infections. Interestingly, it has been reported that NRF2-activating molecules are able to inhibit inflammatory responses and the replication of respiratory viruses [29,43]. Our findings are consistent with the hypothesis that NRF2 antioxidant response may be effective as a critical control mechanism in reducing production of IFN-stimulated genes during RSV infections in the upper respiratory tract, and suggest the possible use of NRF2 activators to counteract viral replication and ameliorate the clinical course in RSV-infected children.

## Figures and Tables

**Table 1:** Patients’ data and relative gene expression of antioxidant and IFN-related genes from 85 RSV- and HRV-positive respiratory samples.

Patients (N = 85)	RSV (N = 63)	HRV (N = 22)	*p*-Value ^a^
Demographic and clinical data			
Male/female	36/27	16/6	0.217
Age in months ^b^	4.91 (5.25)	4.98 (4.68)	0.960
PICU admission	7 (11.1%)	0 (0.0%)	0.182
Severity score (1–3): N (%) ^c^	17/51 (33.3%)	12/19 (63.2%)	0.033
Severity score (4–5): N (%) ^c^	18/51 (35.3%)	6/19 (31.6%)	
Severity score (6–8): N (%) ^c^	16/51 (31.4%)	1/19 (5.2%)	
Gene expression ^d^			
NRF2	1.77 (1.15)	2.23 (1.22)	0.01
G6PD	1.01 (1.14)	0.74 (0.77)	0.670
HO1	0.14 (0.43)	0.49 (0.53)	0.007
NQO1	0.26 (0.74)	0.63 (1.39)	0.354
IFNBeta	−0.37 (0.85)	−0.09 (0.88)	0.236
IFNL1	−0.49 (1.07)	−0.56 (1.21)	0.700
IFNL2	−0.08 (1.06)	−0.08 (1.16)	0.779
IFNL3	0.54 (0.89)	0.44 (1.33)	0.627
ISG15	2.39 (1.21)	1.64 (0.75)	0.006
ISG56	1.32 (1.34)	0.69 (1.05)	0.014
MxA	0.93 (1.07)	0.35 (0.85)	0.028

Abbreviations: RSV, respiratory syncytial virus; HRV, human rhinovirus; PICU, pediatric intensive care unit; NRF2, nuclear factor erythroid 2-related factor 2; G6PD, glucose−6-phosphate dehydrogenase; HO1, heme oxygenase 1; NQO1, NAD(P)H dehydrogenase [quinone] 1; IFN: interferon; IFNL1-3: interferon lambda 1–3; ISG15, interferon-stimulated gene 15; MXA, myxovirus resistance A (interferon-inducible gene); ISG56, interferon-stimulated gene 56. ^a^ *p*-values were calculated with Pearson’s chi-square test for discrete variables: Mann–Whitney tests were used to compare quantitative data. ^b^ mean (standard deviation). ^c^ data were available for 70 (51 RSV- and 19 HRV-positive) bronchiolitis cases. ^d^ Transcript levels of NRF2, G6PD, HO1, and NQO1 genes were determined by qPCR reactions with the SensiFAST SYBR NO-ROX Kit; the other target gene levels were calculated by the comparative Ct method (2^−ΔCt^) with the housekeeping gene β-glucuronidase/GUS. All expression values were log-transformed and are reported as median and interquartile range (IQR).

## Data Availability

The datasets containing all data analyzed, supporting results of this study, will be made available by the authors, without undue reservation.
